# Peptide data on the disulfide bond analysis of baculovirus produced Pfs25 by LC-MSMS

**DOI:** 10.1016/j.dib.2018.03.034

**Published:** 2018-03-12

**Authors:** Shwu-Maan Lee, Jordan L. Plieskatt, C. Richter King

**Affiliations:** PATH Malaria Vaccine Initiative (MVI), 455 Massachusetts Avenue NW, Suite 1000, Washington, DC 20001-2621, USA

**Keywords:** Pfs25, Disulfide, Mass spectrometry, Malaria, LC-MSMS

## Abstract

This article contains the peptide data obtained while performing disulfide bond mapping of the recombinant *Plasmodium* falciparum protein, Pfs25, produced from the baculovirus expression system. Pfs25 is a malaria transmission-blocking vaccine candidate, with a compact and complex structure including 22 cysteines. This supplementary data is related to the research “Disulfide bond mapping of Pfs25, a recombinant malaria transmission blocking vaccine candidate” (Lee et al., 2018) [1]. In brief, Pfs25 was digested with trypsin/Lys-C and derived peptides separated by High Performance Liquid Chromatography (HPLC) and analyzed by mass spectrometry (MS) by MS^E^ fragmentation. The theoretical peptides and their respective masses along with disulfide bond locations with linked peptides are presented here alongside the mass spectrometry analysis. The raw mass spectrometry data is made available through the Mass Spectrometry Interactive Virtual Environment (MassIVE) with identifier: MSV000081982.

**Specifications Table**

**Table:** 

Subject area	Chemistry, Biology,
More specific subject area	Disulfide bond analysis by liquid chromatography and mass spectrometric analysis
Type of data	Tables, figures
How data was acquired	Data was generated using liquid chromatography (Waters 2695 Separations Module and Waters 2489 UV/Vis Detector) and mass spectroscopy (Waters QTOF Premier mass spectrometer)
Data format	Collated data from analysis with Waters BiopharmaLynx 1.3 and MassLynx
Experimental factors	Recombinant Pfs25 digested with 20 µg of trypsin/Lys-C at 37 °C overnight and subsequent further digestion by additional 20 µg of trypsin/Lys-C for 3–4 hours at 37 °C
Experimental features	Identification of the proper pairing of 11 disulfide bonds in Pfs25 through digestion of peptides and LC-MS/MS
Data source location	Mass spectrometry data acquired in Middleton, WI, USA
Data accessibility	Data is provided within this article and RAW MS files have been deposited in the Mass Spectrometry Interactive Virtual Environment (MassIVE) with identifier: MSV000081982 (ftp://massive.ucsd.edu/MSV000081982). MassIVE is a member of the ProteomeXchange Consortium

**Value of the data**

• The derived peptides and mass spectrometry data is provided here for further details from the disulfide bond analysis of Pfs25.
• The disulfide bond locations and linked peptides are discussed alongside the mass spectrometry analysis here and data made accessible to the scientific community.
• Pfs25 is a compact and complex 17.9 kDa protein with 22 cysteines (11 disulfide bonds) that has presented difficulty in prior disulfide bond analysis
• A method was developed to map the disulfide bonds of a complex and compact protein, which may be applicable to other proteins, an important step in recombinant protein development for vaccines.

## 1 Data

The Pfs25 disulfide bond mapping peptides are discussed in further detail in this manuscript to further support the elucidation of disulfide bonds of Pfs25 as discussed in [1]. Further, the mass spectrometry RAW files have been deposited in the Mass Spectrometry Interactive Virtual Environment (MassIVE). Theoretical peptides, produced from Trypsin/Lys-C digestion of Pfs25, are presented in Table 1.

**Table 1:** Theoretical Fragments for Trypsin/Lys-C Digestion of Pfs25.

Peptides	Position	Peptide Label	Theoretical Mass (Da)
DAK	1–3	T1	332.17
VTVDTVCK	4–11	T2	863.44
R	12-12	T3	174.11
GFLIQMSGHLECK	13–25	T4	1461.71
CENDLVLVNEETCEEK	26–41	T5	1865.80
VLK	42–44	T6	358.26
CDEK	45–48	T7	493.18
TVNKPCGDFSK	49–59	T8	1194.57
CIK	60–62	T9	362.20
IDGNPVSYACK	63–73	T10	1165.54
CNLGYDMVNNVCIPNECK	74–91	T11	2027.86
QVTCGNGK	92–99	T12	805.38
CILDTSNPVK	100–109	T13	1088.55
TGVCSCNIGK	110–119	T14	980.44
VPNVQDQNK	120–128	T15	1040.53
CSK	129–131	T16	336.15
DGETK	132–136	T17	548.24
CSLK	137–140	T18	449.23
CLK	141–143	T19	362.20
ENETCK	144–149	T20	722.29
AVDGIYK	150–156	T21	764.41
CDCK	157–160	T22	467.15
DGFIIDQESSICTHHHHHH	161–179	T23	2248.98

Utilizing Biopharmalynx 1.3 the mass spectral data was analyzed and compared to the theoretical peptides to obtain the localization of the 11 disulfide bonds present in the recombinant Pfs25. The disulfide bond locations and linked peptides (including theoretical and observed) masses are presented in Table 2. Each disulfide bond (referenced by nomenclature SS#) is further presented with the peptide information and mass spectrometry (MS) and MSMS data obtained during the analysis in the subsequent figures and tables presented in this manuscript.

**Table 2:** Disulfide bond locations for Pfs25 including theoretical and observed masses of fragments.

**Disulfide Bond**	**Linked Cysteines**	**Tryptic Peptide Label**	**Theoretical Mass (Da)**	**Observed Mass (Da)**	**Mass Error (ppm)**
SS1	Cys_10_-Cys_24_	T2+T4	2323.1375	2323.1152	9.6
SS2	Cys_26_-Cys_38_	T5	1863.7866	1863.7752	6.1
SS3	Cys_45_-Cys_60_	T7+T9	853.3674	853.3656	2.1
SS4	Cys_54_-Cys_72_	T8+T10	2358.0670	2358.0799	5.5
SS5	Cys_74_-Cys_85_	T11+T13	3112.3796	3112.3534	8.4
SS6	Cys_90_-Cys_100_				
SS7	Cys_95_-Cys_113_	T12+T14+T16	2117.9326	2117.9182	6.8
SS8	Cys_115_-Cys_129_				
SS9	Cys_137_-Cys_148_	T18+T20	1169.5056	1169.4970	7.3
SS10	Cys_141_-Cys_157_	T19+T22+T23	3074.3003	3074.2795	6.8
SS11	Cys_159_-Cys_172_				

## 2 Disulfide bond SS1

**Fig. 1:**
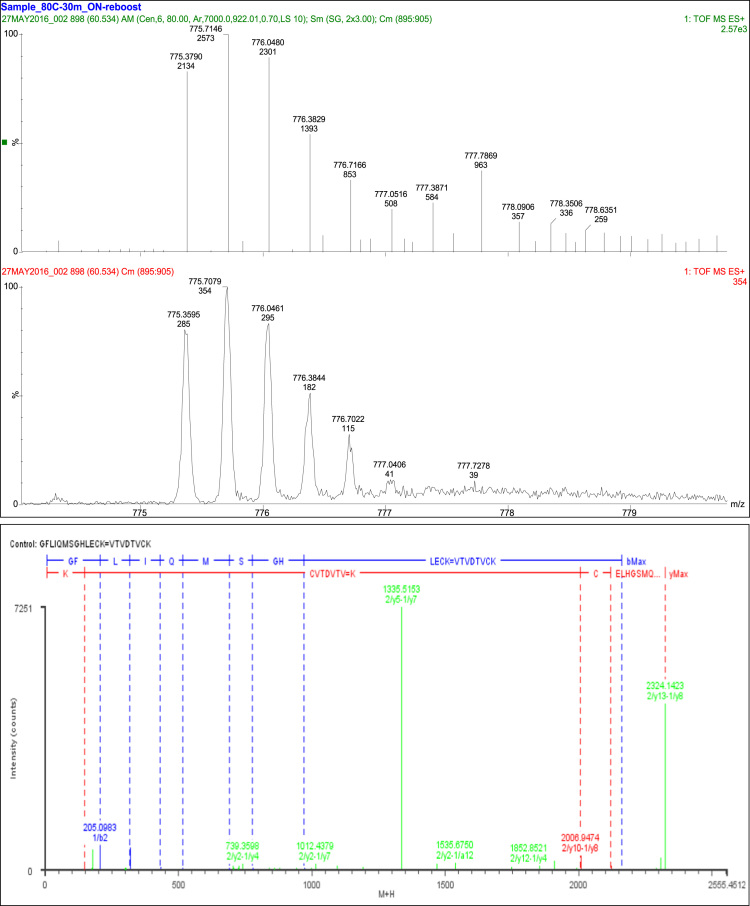
Disulfide bond SS1.

A total of 30 fragments were observed, with 20 fragment ions of this peptide consistent with the linkage of Cys_10_ and Cys_24_. The remaining ten fragment ions were consistent with constituent peptides (Table 3, Fig. 1).

**Table 3:** Disulfide bond SS1 (Cys10-Cys24) peptides.

		**Assignment**	**Theoretical Mass (Da)**	**Observed Mass (Da)**	**Mass Error (Da)**	**Intensity (counts)**	**Identification**
**30 Fragments**	Constituent Peptides 10 Fragments	1/a2	173.129	173.0568	0.0722	38	VT
		1/b5	516.267	516.2606	0.0063	69	VTVDT
		1/y1	147.1133	147.0415	0.0718	19	K
		2/a2	177.1028	177.1026	0.0002	567	GF
		2/b2	205.0977	205.0983	−0.0006	691	GF
		2/b3	318.1818	318.1831	−0.0013	634	GFL
		2/b4	431.2658	431.1857	0.0801	82	GFLI
		2/b6	690.3649	690.3612	0.0037	40	GFLIQM
		2/b7	777.3969	777.4837	−0.0868	34	GFLIQMS
		2/b9	971.4773	971.4425	0.0348	52	GFLIQMSGH
					
	Cys10 and Cys24 20 Fragments	1/b7-2/y3	1094.486	1094.477	0.0095	103	VTVDTVC=ECK
		1/b7-2/y9	1747.782	1747.827	−0.0454	86	VTVDTVC=QMSGHLECK
		1/y2-2/a12	1535.717	1535.675	0.0422	196	CK=GFLIQMSGHLEC
		1/y2-2/y4	739.3483	739.3598	−0.0115	163	CK=LECK
		1/y2-2/y5	876.4072	876.3971	0.0101	68	CK=HLECK
		1/y2-2/z4	722.3217	722.2222	0.0995	26	CK=LECK
		1/y3-2/y3	725.3326	725.3351	−0.0025	101	VCK=ECK
		1/y3-2/y4	838.4167	838.3221	0.0946	56	VCK=LECK
		1/y4-2/y12	1852.912	1852.852	0.0603	119	TVCK=FLIQMSGHLECK
		1/y5-2/y3	941.4072	941.3948	0.0125	50	DTVCK=ECK
		1/y5-2/y5	1191.55	1191.505	0.0454	74	DTVCK=HLECK
		1/y5-2/y7	1335.604	1335.515	0.0884	7251	DTVCK=SGHLECK
		1/y5-2/y8	1466.644	1466.662	−0.0175	177	DTVCK=MSGHLECK
		1/y7-2/y10	1907.903	1907.823	0.0797	268	TVDTVCK=IQMSGHLECK
		1/y7-2/y2	1012.481	1012.438	0.0428	181	TVDTVCK=CK
		1/y8-2/y10	2006.971	2006.947	0.0239	390	VTVDTVCK=IQMSGHLECK
		1/y8-2/y11	2120.055	2120.046	0.0098	112	VTVDTVCK=LIQMSGHLECK
		1/y8-2/y13	2324.145	2324.142	0.0029	4580	VTVDTVCK=GFLIQMSGHLECK
		1/y8-2/z10	1989.945	1989.963	−0.0178	63	VTVDTVCK=IQMSGHLECK
		1/z7-2/y2	995.4542	995.5009	−0.0468	47	TVDTVCK=CK

## 3 Disulfide bond SS2

**Fig. 2:**
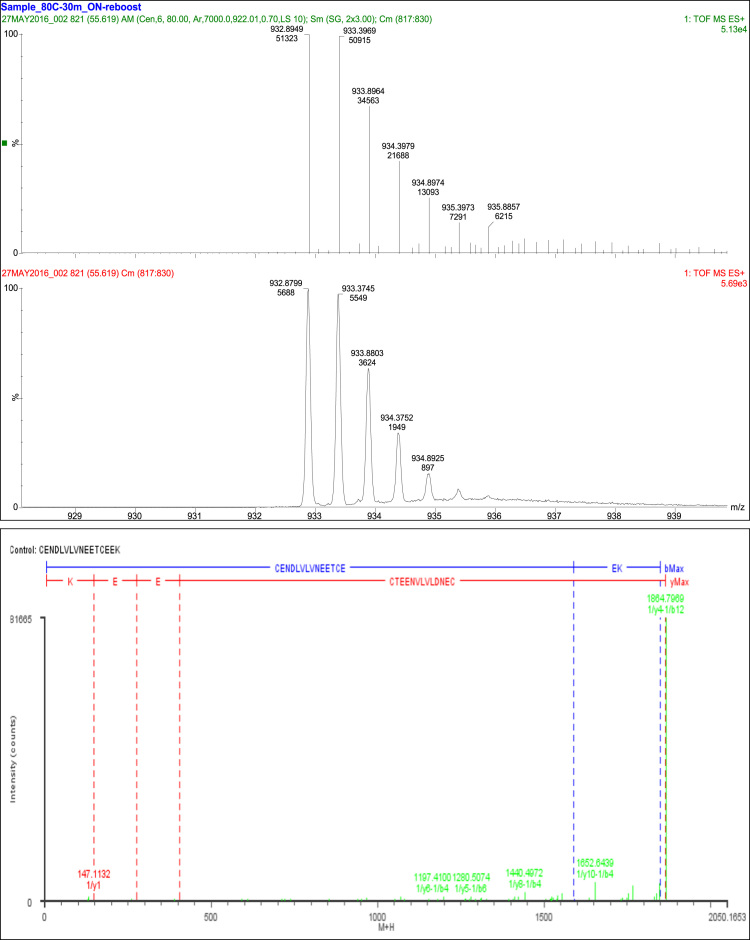
Disulfide bond SS2.

A total of 39 fragment ions of this peptide were observed with 36 fragment ions consistent with the linkage of Cys_26_ and Cys_38_. Three additional fragments were consistent with constituent peptides (Table 4, Fig. 2).

**Table 4:** Disulfide bond SS2 (Cys26-Cys38) peptides.

		**Assignment**	**Theoretical Mass (Da)**	**Observed Mass (Da)**	**Mass Error (Da)**	**Intensity (counts)**	**Identification**
**39 Fragments**	Const. Pep.3 Fragments	1/y1	147.1133	147.1132	0.0001	2129	K
		1/y2	276.1559	276.159	−0.0031	1082	EK
		1/y3	405.1985	405.1972	0.0013	825	EEK
					
	Cys26 and Cys38 36 Fragments	1/b14	1589.646	1589.614	0.0321	335	CENDLVLVNEETCE (Internal)
		1/y10-1/a3	1509.62	1509.584	0.0366	87	LVNEETCEEK=CEN
		1/y10-1/b3	1537.615	1537.644	−0.0291	246	LVNEETCEEK=CEN
		1/y10-1/b4	1652.642	1652.644	−0.002	5544	LVNEETCEEK=CEND
		1/y10-1/b5	1765.726	1765.722	0.0043	4497	LVNEETCEEK=CENDL
		1/y11-1/b4	1751.71	1751.709	0.0017	2109	VLVNEETCEEK=CEND
		1/y12-1/b1	1506.682	1506.587	0.0948	66	LVLVNEETCEEK=C
		1/y12-1/b2	1635.725	1635.631	0.0941	586	LVLVNEETCEEK=CE
		1/y4-1/a12	1836.8	1836.787	0.0127	2176	CEEK=CENDLVLVNEET
		1/y4-1/a2	710.249	710.2508	−0.0018	210	CEEK=CE
		1/y4-1/a6	1151.471	1151.5	−0.0284	109	CEEK=CENDLV
		1/y4-1/a7	1264.555	1264.47	0.0858	499	CEEK=CENDLVL
		1/y4-1/b1	609.2012	609.2489	−0.0477	393	CEEK=C
		1/y4-1/b10	1634.704	1634.629	0.0752	999	CEEK=CENDLVLVNE
		1/y4-1/b12	1864.794	1864.797	−0.0024	81665	CEEK=CENDLVLVNEET
		1/y4-1/b2	738.2438	738.2711	−0.0273	341	CEEK=CE
		1/y4-1/b3	852.2868	852.3066	−0.0198	172	CEEK=CEN
		1/y4-1/b4	967.3137	967.3153	−0.0016	992	CEEK=CEND
		1/y4-1/b5	1080.398	1080.422	−0.024	317	CEEK=CENDL
		1/y4-1/b6	1179.466	1179.442	0.024	380	CEEK=CENDLV
		1/y4-1/b7	1292.55	1292.497	0.053	258	CEEK=CENDLVL
		1/y4-1/b8	1391.619	1391.581	0.0372	224	CEEK=CENDLVLV
		1/y5-1/a5	1153.451	1153.433	0.0179	274	TCEEK=CENDL
		1/y5-1/b4	1068.362	1068.366	−0.004	1172	TCEEK=CEND
		1/y5-1/b6	1280.514	1280.507	0.0065	1279	TCEEK=CENDLV
		1/y5-1/b8	1492.666	1492.607	0.059	72	TCEEK=CENDLVLV
		1/y6-1/a6	1381.562	1381.568	−0.0067	109	ETCEEK=CENDLV
		1/y6-1/b4	1197.404	1197.41	−0.0061	1266	ETCEEK=CEND
		1/y6-1/b5	1310.488	1310.488	0.0001	885	ETCEEK=CENDL
		1/y6-1/b6	1409.557	1409.556	0.0009	1232	ETCEEK=CENDLV
		1/y7-1/a1	940.3392	940.3529	−0.0137	271	EETCEEK=C
		1/y7-1/b4	1326.447	1326.445	0.0021	764	EETCEEK=CEND
		1/y7-1/b6	1538.599	1538.576	0.0226	1457	EETCEEK=CENDLV
		1/y8-1/a5	1525.579	1525.593	−0.014	1104	NEETCEEK=CENDL
		1/y8-1/b4	1440.49	1440.497	−0.0077	2676	NEETCEEK=CEND
		1/y8-1/b5	1553.574	1553.575	−0.0016	2348	NEETCEEK=CENDL

## 4 Disulfide bond SS3

**Fig. 3:**
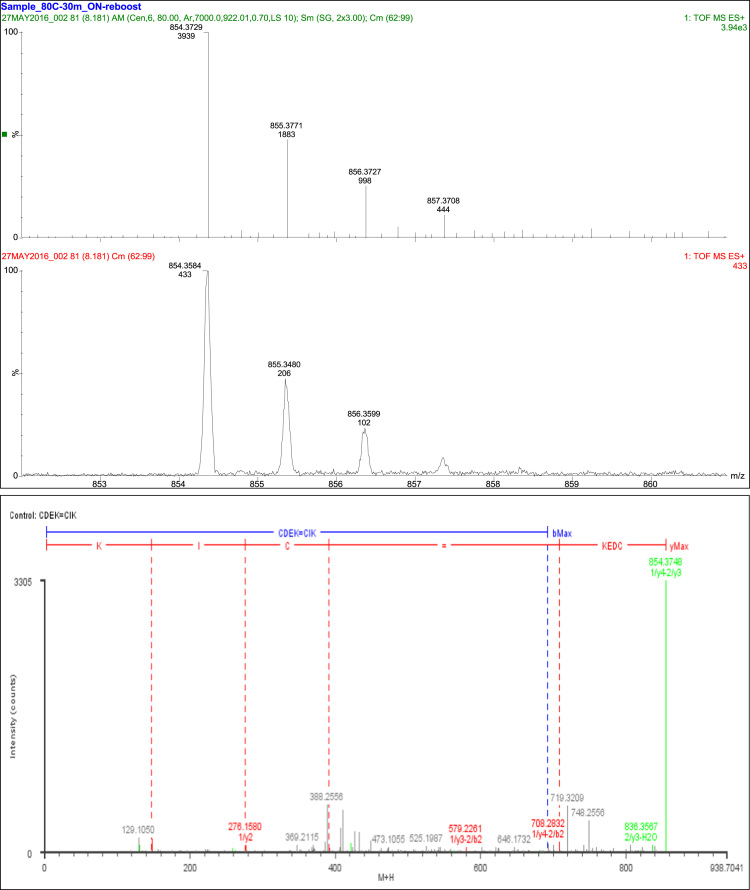
Disulfide bond SS3.

A total of nine fragments were observed, with six fragment ions of this peptide consistent with the linkage of Cys_45_ and Cys_60_. Three fragment ions were consistent with constituent peptides (Table 5, Fig. 3).

**Table 5:** Disulfide bond SS3 (Cys45-Cys60) peptides.

		**Assignment**	**Theoretical Mass (Da)**	**Observed Mass (Da)**	**Mass Error (Da)**	**Intensity (counts)**	**Identification**
**9 Fragments**	Const. Pep.3 Fragments	1/y1	147.1133	147.115	−0.0017	163	K
		1/y2	276.1559	276.158	−0.002	84	EK
		1/y3	391.1829	391.2075	−0.0246	47	DEK
	Cys45 and Cys60 6 Fragments	1/a1–2/a2	262.1048	262.0183	0.0865	22	C=CI
		1/b1–2/a3	421.0852	421.1291	−0.0439	117	C=CIK
		1/b2-2/y3	579.2271	579.2261	0.001	67	CD=CIK
		1/y4-2/a2	680.2748	680.2768	−0.002	23	CDEK=CI
		1/y4-2/b2	708.2697	708.2832	−0.0135	126	CDEK=CI
		1/y4-2/y3	854.3752	854.3748	0.0004	3305	CDEK=CIK

## 5 Disulfide bond SS4

**Fig. 4:**
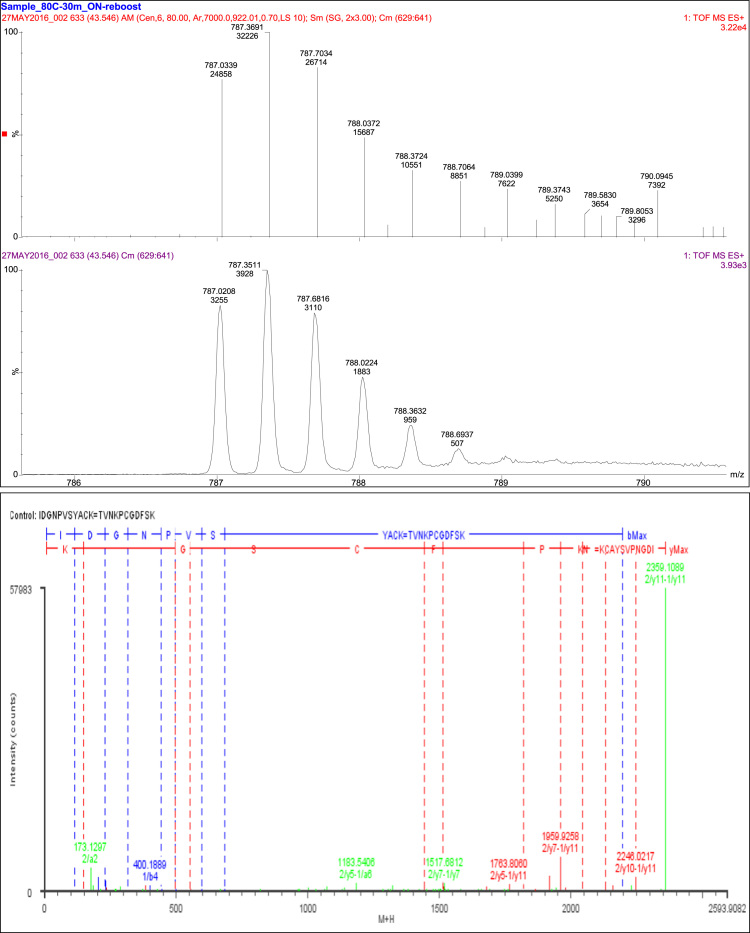
Disulfide bond SS4.

A total of 68 fragments were observed, with 45 fragment ions of this peptide consistent with the linkage of T8 to T10 through Cys_54_ and Cys_72_. The remaining 23 fragments were consistent with constituent peptides (Table 6, Fig. 4).

**Table 6:** Disulfide bond SS4 (Cys54-Cys72) peptides.

		**Assignment**	**Theoretical Mass (Da)**	**Observed Mass (Da)**	**Mass Error (Da)**	**Intensity (counts)**	**Identification**
**68 Fragments**	Constituent Peptides 23 Fragments	1/a2	173.129	173.1297	−0.0007	4507	TV
		1/a3	287.1719	287.0969	0.075	897	TVN
		1/a4	415.2669	415.2224	0.0445	28	TVNK
		1/b2	201.1239	201.1235	0.0004	2754	TV
		1/b3	315.1668	315.0896	0.0772	51	TVN
		1/b4	443.2618	443.2626	−0.0008	248	TVNK
		1/y1	147.1133	147.0623	0.051	16	K
		1/y2	234.1454	234.1455	−0.0001	700	SK
		1/y3	381.2138	381.2014	0.0124	1146	FSK
		1/y4	496.2407	496.2404	0.0003	153	DFSK
		1/y5	553.2622	553.2604	0.0018	564	GDFSK
		2/a3	258.1454	258.1545	−0.0092	78	IDG
		2/a4	372.1883	372.1927	−0.0044	300	IDGN
		2/a5	469.2411	469.2278	0.0132	76	IDGNP
		2/a6	568.3094	568.3065	0.0029	91	IDGNPV
		2/a8	818.4048	818.3653	0.0396	129	IDGNPVSY
		2/b1	114.0919	114.041	0.0509	18	I
		2/b2	229.1188	229.119	−0.0002	1962	ID
		2/b3	286.1403	286.1415	−0.0012	727	IDG
		2/b4	400.1832	400.1889	−0.0057	1203	IDGN
		2/b5	497.236	497.2373	−0.0013	252	IDGNP
		2/b6	596.3044	596.3082	−0.0038	205	IDGNPV
		2/b7	683.3364	683.3206	0.0158	211	IDGNPVS
					
	Cys54 and Cys72 45 Fragments	1/a6-2/y5	1183.5604	1183.541	0.0198	1608	TVNKPC=SYACK
		1/a6-2/y7	1379.6815	1379.604	0.0773	143	TVNKPC=PVSYACK
		1/a8-2/y7	1551.73	1551.666	0.0636	108	TVNKPCGD=PVSYACK
		1/a9-2/b10	1951.8682	1951.823	0.0454	93	TVNKPCGDF=IDGNPVSYAC
		1/b10-2/a10	2038.9003	2038.99	−0.09	43	TVNKPCGDFS=IDGNPVSYAC
		1/b6-2/y3	961.4599	961.4065	0.0534	124	TVNKPC=ACK
		1/b6-2/y8	1521.7194	1521.674	0.0449	161	TVNKPC=NPVSYACK
		1/b7-2/y7	1464.6979	1464.675	0.0228	47	TVNKPCG=PVSYACK
		1/b8-2/y10	1865.8162	1865.827	−0.0106	68	TVNKPCGD=DGNPVSYACK
		1/b8-2/y11	1978.9003	1978.891	0.0092	579	TVNKPCGD= IDGNPVSYACK
		1/b8-2/y2	1062.4712	1062.441	0.0303	364	TVNKPCGD=CK
		1/b8-2/y6	1482.6721	1482.651	0.021	137	TVNKPCGD=VSYACK
		1/b8-2/y7	1579.7249	1579.69	0.0347	456	TVNKPCGD=PVSYACK
		1/b9-2/y5	1530.6721	1530.667	0.0054	180	TVNKPCGDF=SYACK
		1/b9-2/y7	1726.7932	1726.741	0.0527	66	TVNKPCGDF=PVSYACK
		1/y11-2/y10	2246.0222	2246.022	0.0005	2740	TVNKPCGDFSK=DGNPVSYACK
		1/y11-2/y11	2359.1062	2359.109	−0.0027	57983	TVNKPCGDFSK= IDGNPVSYACK
		1/y11-2/y2	1442.6771	1442.674	0.0034	677	TVNKPCGDFSK=CK
		1/y11-2/y3	1513.7142	1513.711	0.0028	849	TVNKPCGDFSK=ACK
		1/y11-2/y4	1676.7776	1676.777	0.0009	999	TVNKPCGDFSK=YACK
		1/y11-2/y5	1763.8097	1763.806	0.0037	1320	TVNKPCGDFSK=SYACK
		1/y11-2/y6	1862.8781	1862.893	−0.0144	201	TVNKPCGDFSK=VSYACK
		1/y11-2/y7	1959.9308	1959.926	0.005	6462	TVNKPCGDFSK=PVSYACK
		1/y11-2/y9	2130.9951	2131.013	−0.0173	418	TVNKPCGDFSK=GNPVSYACK
		1/y11-2/z10	2228.9956	2228.999	−0.0037	1038	TVNKPCGDFSK=DGNPVSYACK
		1/y6-2/a10	1645.699	1645.775	−0.0762	368	CGDFSK=IDGNPVSYAC
		1/y6-2/y11	1819.7994	1819.795	0.0044	57	CGDFSK= IDGNPVSYACK
		1/y6-2/y4	1137.4708	1137.472	−0.0007	738	CGDFSK=YACK
		1/y6-2/y7	1420.624	1420.613	0.0114	404	CGDFSK=PVSYACK
		1/y7-2/b10	1770.7467	1770.722	0.0243	43	PCGDFSK= IDGNPVSYAC
		1/y7-2/y11	1916.8523	1916.848	0.0039	3031	PCGDFSK= IDGNPVSYACK
		1/y7-2/y2	1000.4232	1000.424	−0.0004	627	PCGDFSK=CK
		1/y7-2/y3	1071.4603	1071.461	−0.0007	825	PCGDFSK=ACK
		1/y7-2/y5	1321.5557	1321.558	−0.0024	1193	PCGDFSK=SYACK
		1/y7-2/y7	1517.6769	1517.681	−0.0043	1667	PCGDFSK=PVSYACK
		1/y7-2/y9	1688.7412	1688.747	−0.0057	547	PCGDFSK=GNPVSYACK
		1/y8-2/y11	2044.9471	2044.929	0.0182	64	KPCGDFSK= IDGNPVSYACK
		1/y8-2/y2	1128.5182	1128.502	0.0162	491	KPCGDFSK=CK
		1/y8-2/y4	1362.6187	1362.648	−0.0288	120	KPCGDFSK=YACK
		1/y8-2/y5	1449.6506	1449.647	0.0038	253	KPCGDFSK=SYACK
		1/y8-2/y8	1759.8147	1759.8	0.0151	186	KPCGDFSK=NPVSYACK
		1/y9-2/y11	2158.9902	2158.979	0.0112	1071	NKPCGDFSK=IDGNPVSYACK
		1/y9-2/y3	1313.5981	1313.58	0.0184	279	NKPCGDFSK=ACK
		1/y9-2/y4	1476.6615	1476.644	0.0173	144	NKPCGDFSK=YACK
		1/z7-2/y2	983.3967	983.4453	−0.0486	113	PCGDFSK=CK

## 6 Disulfide bonds SS5 and SS6

**Fig. 5:**
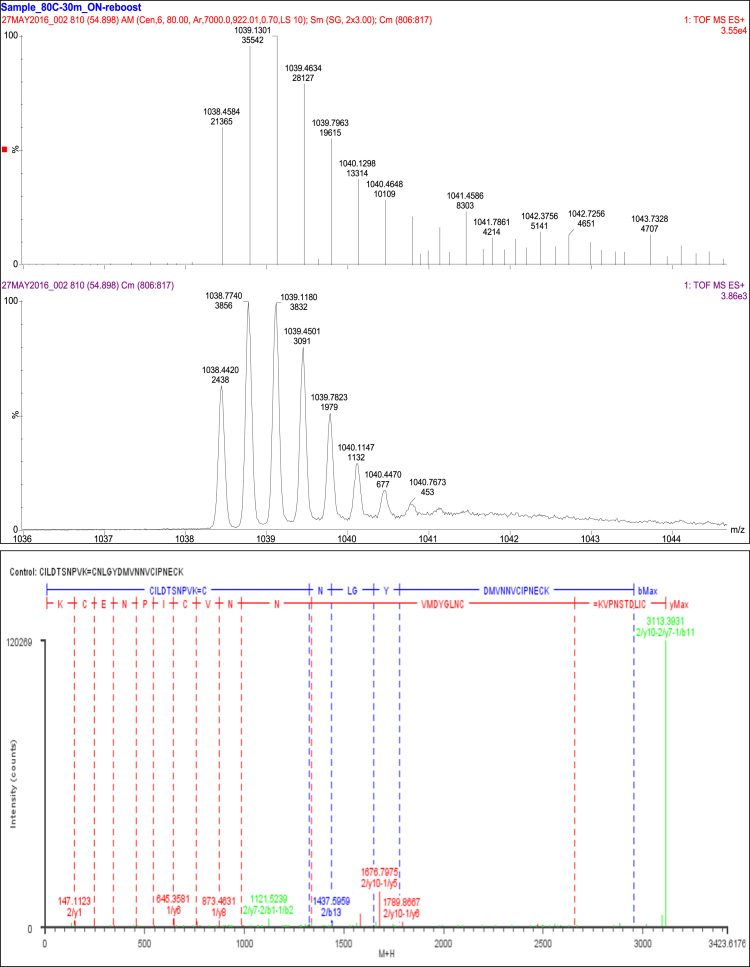
Disulfide bonds SS5 and SS6.

A total of 90 fragments were observed and four fragment ions (1/b12, 1/b13, 1/b15, and 1/b16) were consistent with an internal disulfide bond linkage between Cys_74_ and Cys_85._ Thirty-three fragment ions were consistent with the linkage of Cys_90_ and Cys_100_. An additional 44 fragments of this peptide were consistent with the combined linkages of Cys_74_ to Cys_85_ and Cys_90_ to Cys_100_ and nine fragments consistent with constituent peptides (Table 7, Fig. 5).

**Table 7:** Disulfide bond SS5 (Cys74-Cys85) and SS6 (Cys90-Cys100) peptides.

		**Assignment**	**Theoretical Mass (Da)**	**Observed Mass (Da)**	**Mass Error (Da)**	**Intensity (counts)**	**Identification**
**90 Fragments**	Cys74 and Cys85 4 Fragments	1/b12	1324.513	1324.516	−0.0031	1267	CNLGYDMVNNVC (Internal)
		1/b13	1437.597	1437.596	0.0006	3004	CNLGYDMVNNVCI (Internal)
		1/b15	1648.692	1648.71	−0.0175	311	CNLGYDMVNNVCIPN (Internal)
		1/b16	1777.735	1777.728	0.007	105	CNLGYDMVNNVCIPNE (Internal)
					
	Constituent Peptides 9 Fragments	1/y1	147.1133	147.1123	0.001	2902	K
		2/y2	246.1818	246.1842	−0.0024	789	VK
		2/y3	343.2345	343.1509	0.0836	258	PVK
		2/y4	457.2774	457.2771	0.0003	855	NPVK
		2/y5	544.3095	544.3134	−0.0039	711	SNPVK
		2/y6	645.3572	645.3581	−0.0009	3696	TSNPVK
		2/y7	760.3841	760.3795	0.0046	2330	DTSNPVK
		2/y8	873.4681	873.4631	0.0051	2651	LDTSNPVK
		2/y9	986.5522	986.5494	0.0029	381	ILDTSNPVK
					
	Cys90-Cys100 33 fragments	1/a17-2/a7	2569.065	2569.078	−0.0127	433	CNLGYDMVNNVCIPNEC=CILDTSN
		1/y18-2/b2	2240.927	2240.983	−0.0564	114	CNLGYDMVNNVCIPNECK=CI
		1/y18-2/b4	2469.038	2469.068	−0.03	1446	CNLGYDMVNNVCIPNECK=CILD
		1/y18-2/b6	2657.118	2657.106	0.012	90	CNLGYDMVNNVCIPNECK=CILDTS
		1/y2-2/a8	1063.492	1063.406	0.0859	167	CK=CILDTSNP
		1/y2-2/b1	351.1161	351.204	−0.0879	78	CK=C
		1/y2-2/b2	464.2001	464.2095	−0.0094	359	CK=CI
		1/y2-2/b3	577.2842	577.284	0.0002	142	CK=CIL
		1/y2-2/b4	692.3112	692.2997	0.0115	322	CK=CILD
		1/y2-2/b7	994.4338	994.4278	0.006	837	CK=CILDTSN
		1/y2-2/y10	1336.661	1336.634	0.027	664	CK=CILDTSNPVK
		1/y3-2/b1	480.1587	480.1505	0.0081	107	ECK=C
		1/y3-2/b4	821.3538	821.3419	0.0118	460	ECK=CILD
		1/y3-2/b5	922.4014	922.3771	0.0244	283	ECK=CILDT
		1/y3-2/b8	1220.529	1220.519	0.0098	340	ECK=CILDTSNP
		1/y3-2/b9	1319.598	1319.584	0.0138	987	ECK=CILDTSNPV
		1/y4-2/a1	566.2067	566.2282	−0.0215	232	NECK=C
		1/y4-2/a2	679.2908	679.3111	−0.0203	202	NECK=CI
		1/y4-2/a7	1209.524	1209.502	0.0221	927	NECK=CILDTSN
		1/y4-2/b3	820.3697	820.3667	0.003	154	NECK=CIL
		1/y4-2/b4	935.3967	935.3924	0.0043	481	NECK=CILD
		1/y4-2/b5	1036.444	1036.448	−0.0039	191	NECK=CILDT
		1/y4-2/b8	1334.572	1334.572	0	1655	NECK=CILDTSNP
		1/y4-2/y10	1579.746	1579.675	0.0706	5505	NECK=CILDTSNPVK
		1/y5-2/b8	1431.625	1431.599	0.0256	644	PNECK=CILDTSNP
		1/y5-2/y10	1676.799	1676.798	0.0012	15020	PNECK=CILDTSNPVK
		1/y6-2/a1	776.3435	776.3327	0.0108	842	IPNECK=C
		1/y6-2/a6	1305.618	1305.539	0.0798	217	IPNECK=CILDTS
		1/y6-2/b2	917.4225	917.4044	0.0181	1033	IPNECK=CI
		1/y6-2/b3	1030.507	1030.413	0.0941	390	IPNECK=CIL
		1/y6-2/y10	1789.883	1789.867	0.0161	2137	IPNECK=CILDTSNPVK
		1/z4-2/y10	1562.72	1562.661	0.0585	2069	NECK=CILDTSNPVK
		1/z5-2/y10	1659.772	1659.854	−0.0822	2401	PNECK=CILDTSNPVK
					
	Cys74 to Cys85 and Cys90 to Cys100 44 Fragments	1/y10-1/a1–2/a2	1392.59	1392.579	0.0105	789	NVCIPNECK=C=CI
		1/y10-2/b9-1/a5	2594.151	2594.122	0.0295	85	NVCIPNECK=CILDTSNPV=CNLGY
		1/y11-1/a4-2/a4	2003.918	2003.83	0.0873	148	VNNVCIPNECK=CNLG=CILD
		1/y11-1/b4-2/b1	1718.712	1718.686	0.0265	115	VNNVCIPNECK=CNLG=C
		1/y11-1/b6-2/b6	2526.077	2525.993	0.084	186	VNNVCIPNECK=CNLGYD=CILDTS
		1/y12-2/a2-1/a5	2069.91	2069.88	0.0305	554	MVNNVCIPNECK=CI=CNLGY
		1/y12-2/b5-1/a2	2093.895	2093.833	0.0625	298	MVNNVCIPNECK=CILDT=CN
		1/y13-1/b1–2/a9	2492.075	2492.059	0.0166	490	DMVNNVCIPNECK=C=CILDTSNPV
		1/y13-1/b2-2/a3	1992.847	1992.854	−0.0063	826	DMVNNVCIPNECK=CN=CIL
		1/y13-2/b3-1/b4	2190.948	2190.909	0.0388	313	DMVNNVCIPNECK=CIL=CNLG
		1/y13-2/b5-1/a2	2208.922	2208.972	−0.0498	102	DMVNNVCIPNECK=CILDT=CN
		1/y14-1/a1–2/a8	2528.075	2527.986	0.0891	572	YDMVNNVCIPNECK=C=CILDTSNP
		1/y14-2/b9-1/a3	2882.266	2882.24	0.0254	1679	YDMVNNVCIPNECK=CILDTSNPV=CNL
		1/y15-1/a1–2/a9	2684.165	2684.074	0.0908	230	GYDMVNNVCIPNECK=C=CILDTSNPV
		1/y15-1/b2-2/b9	2854.198	2854.166	0.0322	311	GYDMVNNVCIPNECK=CN=CILDTSNPV
		1/y7-1/b1–2/b2	1121.425	1121.524	−0.0988	3609	CIPNECK=C=CI
		1/y7-2/a1-1/a2	1066.394	1066.391	0.0034	286	CIPNECK=C=CN
		1/y7-2/a1-1/a8	1744.699	1744.779	−0.0803	1284	CIPNECK=C=CNLGYDMV
		1/y7-2/a2-1/a7	1758.715	1758.619	0.0957	156	CIPNECK=CI=CNLGYDM
		1/y7-2/a3-1/a9	2084.91	2084.817	0.0925	164	CIPNECK=CIL=CNLGYDMVN
		1/y7-2/a5-1/a3	1621.721	1621.642	0.0795	207	CIPNECK=CILDT=CNL
		1/y7-2/a9-1/a11	2911.292	2911.285	0.0068	205	CIPNECK=CILDTSNPV=CNLGYDMVNNV
		1/y7-2/a9-1/a8	2584.138	2584.076	0.062	990	CIPNECK=CILDTSNPV=CNLGYDMV
		1/y7-2/b1-1/a3	1207.473	1207.5	−0.027	508	CIPNECK=C=CNL
		1/y7-2/b1-1/a6	1542.585	1542.666	−0.0806	928	CIPNECK=C=CNLGYD
		1/y7-2/b2-1/b4	1405.574	1405.526	0.0474	125	CIPNECK=CI=CNLG
		1/y7-2/b3-1/b4	1518.658	1518.643	0.015	197	CIPNECK=CIL=CNLG
		1/y7-2/b4-1/a10	2341.975	2341.988	−0.0132	340	CIPNECK=CILD=CNLGYDMVNN
		1/y7-2/b6-1/a10	2530.054	2529.985	0.0696	102	CIPNECK=CILDTS=CNLGYDMVNN
		1/y7-2/b8-1/b3	1975.839	1975.88	−0.0417	245	CIPNECK=CILDTSNP=CNL
		1/y8-1/a1–2/a5	1493.663	1493.629	0.0336	182	VCIPNECK=C=CILDT
		1/y8-2/b9-1/a3	2145.981	2145.931	0.0496	164	VCIPNECK=CILDTSNPV=CNL
		1/y9-1/b1–2/b5	1663.695	1663.662	0.0337	104	NVCIPNECK=C=CILDT
		1/y9-2/b1-1/a6	1755.696	1755.718	−0.0212	658	NVCIPNECK=C=CNLGYD
		1/y9-2/b1-1/b1	1221.453	1221.521	−0.068	782	NVCIPNECK=C=C
		1/y9-2/b8-1/a6	2496.067	2496.051	0.0159	634	NVCIPNECK=CILDTSNP=CNLGYD
		1/y9-2/b9-1/a3	2260.023	2259.99	0.0334	668	NVCIPNECK=CILDTSNPV=CNL
		2/y10-1/y12-1/b2	2665.192	2665.097	0.0942	258	CILDTSNPVK=MVNNVCIPNECK=CN
		2/y10-1/y7-1/b10	3014.319	3014.31	0.0088	1407	CILDTSNPVK=CIPNECK=CNLGYDMVNN
		2/y10-1/y7-1/b11	3113.388	3113.393	−0.0056	120269	CILDTSNPVK=CIPNECK=CNLGYDMVNNV
		2/y10-1/y7-1/b6	2556.124	2556.095	0.0291	1309	CILDTSNPVK=CIPNECK=CNLGYD
		2/y10-1/y9-1/a4	2463.15	2463.093	0.0574	609	CILDTSNPVK=NVCIPNECK=CNLG
		2/y10-1/y9-1/a5	2626.214	2626.114	0.0999	654	CILDTSNPVK=NVCIPNECK=CNLGY
		2/y10-1/y9-1/b3	2434.124	2434.059	0.0645	571	CILDTSNPVK=NVCIPNECK=CNL

## 7 Disulfide bonds SS7 and SS8

**Fig. 6:**
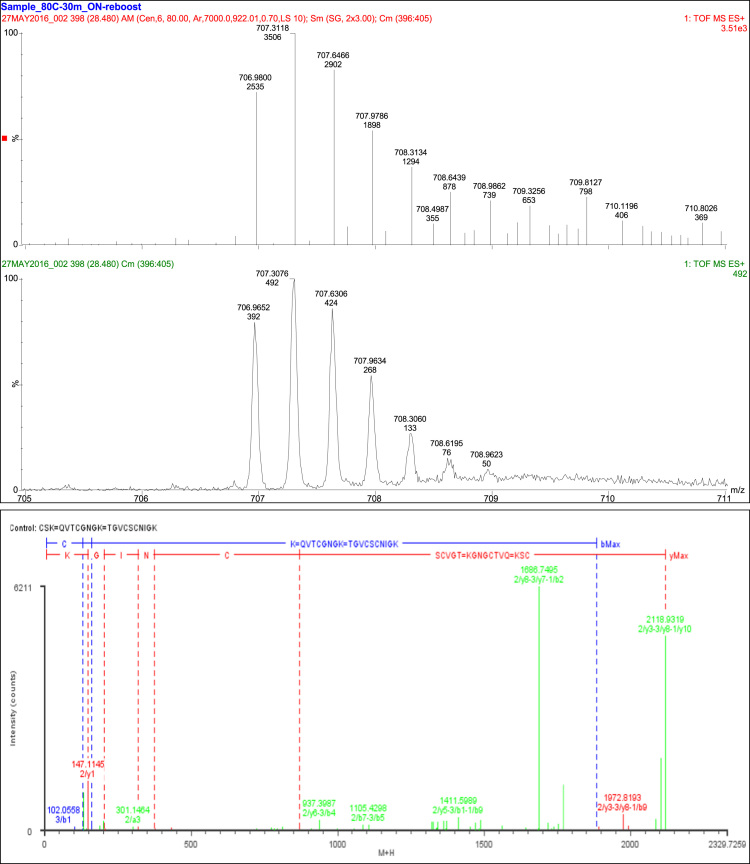
Disulfide bonds SS7 and SS8.

A total of 39 fragments were observed. Three fragment ions (1/b7-2/b5, 1/y6-2/a4, and 1/y6-2/b4) were specific to the linkage between T12 and T14 and confirmed the Cys_95_ to Cys_113_ linkage. Four fragment ions (2/y5-3/b2, 2/y5-3/y3, 2/y6-3/a2, and 2/y6-3/b2) were specific to the linkage between T14 and T16 and confirmed the linkage of Cys_115_ to Cys_129_. A further 21 fragment ions were consistent with the linkage of T12, T14, and T16 and remaining 11 fragments consistent with constituent peptides (Table 8, Fig. 6).

**Table 8:** Disulfide bond SS7 (Cys95-Cys113) and SS8 (Cys115-Cys129) peptides.

		**Assignment**	**Theoretical Mass (Da)**	**Observed Mass (Da)**	**Mass Error (Da)**	**Intensity (counts)**	**Identification**
**39 Fragments**	Constituent Peptides 11 Fragments	1/a2	200.1399	200.141	−0.0011	247	QV
		1/a3	301.1876	301.1464	0.0412	100	QVT
		1/b1	129.0664	129.0653	0.0011	33	Q
		1/y1	147.1133	147.1145	−0.0012	1258	K
		1/y3	318.1777	318.1789	−0.0012	47	NGK
		1/y4	375.1992	375.2	−0.0008	52	GNGK
		2/a2	131.082	131.0274	0.0546	30	T
		2/b1	102.0555	102.0558	−0.0003	107	T
		2/b2	159.077	159.0768	0.0002	140	TG
		2/y2	204.1348	204.0705	0.0643	30	GK
		2/y4	431.2618	431.2624	−0.0006	72	NIGK
					
	Cys95 to Cys113 3 Fragments	1/b7-2/b5	1105.441	1105.43	0.0109	157	QVTCGNG=TGVCS
		1/y6-2/a4	909.3923	909.4301	−0.0378	58	TCGNGK=TGVC
		1/y6-2/b4	937.3871	937.3987	−0.0115	257	TCGNGK=TGVC
					
	Cys115 to Cys129 4 Fragments	2/y5-3/b2	722.2966	722.3025	−0.0059	45	CNIGK=CS
		2/y5-3/y3	868.4021	868.3544	0.0477	103	CNIGK=CSK
		2/y6-3/a2	781.3337	781.4172	−0.0835	21	SCNIGK=CS
		2/y6-3/b2	809.3286	809.3259	0.0027	90	SCNIGK=CS
					
	T12, T14, and T16 linkage 21 Fragments	1/a4-2/a6-3/a1	997.3728	997.3982	−0.0254	39	QVTC=TGVCSC=C
		1/a4-2/a6-3/a2	1084.405	1084.434	−0.0294	146	QVTC=TGVCSC=CS
		1/a4-2/a8-3/b2	1339.527	1339.585	−0.0582	210	QVTC=TGVCSCNI=CS
		1/a4-2/y8-3/y3	1558.685	1558.64	0.0449	111	QVTC=VCSCNIGK=CSK
		1/a5-2/a6-3/a1	1054.394	1054.432	−0.0376	60	QVTCG=TGVCSC=C
		1/a6-2/y8-3/y3	1729.749	1729.66	0.089	51	QVTCGN=VCSCNIGK=CSK
		1/a7-2/a7-3/b1	1367.497	1367.472	0.0244	33	QVTCGNG=TGVCSCN=C
		1/b5-2/a9-3/b2	1481.565	1481.493	0.0713	35	QVTCG=TGVCSCNIG=CS
		1/b6-2/a7-3/b1	1338.47	1338.53	−0.0596	72	QVTCGN=TGVCSCN=C
		1/b6-2/b9-3/y3	1769.708	1769.651	0.0566	1155	QVTCGN=TGVCSCNIG=CSK
		1/y5-2/b6-3/y3	1360.512	1360.561	−0.0487	249	CGNGK=TGVCSC=CSK
		1/y5-2/b9-3/b1	1411.523	1411.599	−0.0762	348	CGNGK=TGVCSCNIG=C
		1/y5-2/y8-3/a1	1371.564	1371.529	0.035	254	CGNGK=VCSCNIGK=C
		1/y5-2/y8-3/b2	1486.591	1486.645	−0.0536	270	CGNGK=VCSCNIGK=CS
		1/y6-2/y10-3/y3	1891.814	1891.761	0.0521	105	TCGNGK=TGVCSCNIGK=CSK
		1/y7-2/b8-3/b2	1641.649	1641.631	0.0188	78	VTCGNGK=TGVCSCNI=CS
		1/y7-2/y10-3/y3	1990.882	1990.798	0.0837	124	VTCGNGK=TGVCSCNIGK=CSK
		1/y7-2/y8-3/b2	1686.707	1686.75	−0.0424	6211	VTCGNGK=VCSCNIGK=CS
		1/y8-2/b8-3/b1	1682.676	1682.723	−0.0466	32	QVTCGNGK=TGVCSCNI=C
		1/y8-2/b9-3/y3	1972.835	1972.819	0.0156	407	QVTCGNGK=TGVCSCNIG=CSK
		1/y8-2/y10-3/y3	2118.94	2118.932	0.0085	4955	QVTCGNGK=TGVCSCNIGK=CSK

## 8 Disulfide bond SS9

**Fig. 7:**
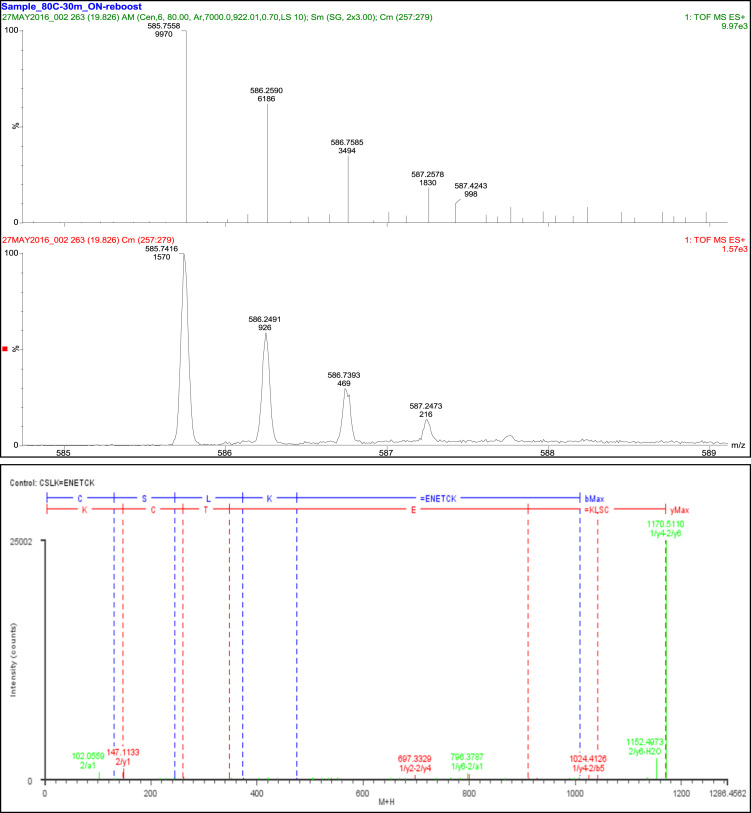
Disulfide bond SS9.

A total of 35 fragment ions were observed with 25 fragment ions of this peptide consistent with the linkage of T18 to T20 through Cys_137_ and Cys_148_. Ten fragments were consistent with constituent peptides (Table 9, Fig. 7).

**Table 9:** Disulfide bond SS9 (Cys137-Cys148) peptides.

		**Assignment**	**Theoretical Mass (Da)**	**Observed Mass (Da)**	**Mass Error (Da)**	**Intensity (counts)**	**Identification**
**35 Fragments**	constituent peptides 10 Fragments	1/y2	260.1974	260.2041	−0.0067	262	LK
		1/y3	347.2294	347.2299	−0.0005	129	SLK
		2/a1	102.0555	102.0559	−0.0004	754	E
		2/a2	216.0984	216.1371	−0.0387	65	EN
		2/a4	446.1887	446.2249	−0.0362	20	ENET
		2/b1	130.0504	130.0469	0.0035	17	E
		2/b2	244.0933	244.0929	0.0004	367	EN
		2/b3	373.1359	373.1376	−0.0017	171	ENE
		2/b4	474.1836	474.1958	−0.0122	88	ENET
		2/y1	147.1133	147.1133	0	1131	K
					
	Cys137 and Cys148 25 Fragments	1/a1–2/y2	323.1212	323.1008	0.0204	15	C=CK
		1/a1–2/y4	553.2114	553.2058	0.0056	53	C=ETCK
		1/a1–2/y5	667.2544	667.2748	−0.0204	59	C=NETCK
		1/a2-2/y2	410.1532	410.1623	−0.0091	36	CS=CK
		1/a2-2/y4	640.2435	640.2652	−0.0217	34	CS=ETCK
		1/a3-2/y3	624.2849	624.2893	−0.0044	27	CSL=CKT
		1/a3-2/y5	867.3705	867.3508	0.0197	89	CSL=NETCK
		1/b1–2/a5	650.1914	650.2377	−0.0463	130	C=ENETC
		1/b2-2/a5	737.2234	737.2545	−0.0311	139	CS=ENETC
		1/b1–2/y2	351.1161	351.1717	−0.0556	207	C=CK
		1/b1–2/y3	452.1638	452.1826	−0.0188	23	C=CKT
		1/b2-2/y2	438.1481	438.1512	−0.0031	139	CS=CK
		1/b2-2/y3	539.1958	539.2008	−0.005	166	CS=CKT
		1/b2-2/y5	782.2813	782.3434	−0.0621	180	CS=NETCK
		1/b3-2/y2	551.2322	551.2324	−0.0002	219	CSL=CK
		1/y4-2/y2	697.3377	697.3329	0.0048	465	CSLK=CK
		1/y4-2/a5	996.413	996.4069	0.0061	130	CSLK=ENETC
		1/y4-2/b5	1024.408	1024.4126	−0.0046	502	CSLK=ENETC
		1/y4-2/y3	798.3854	798.3802	0.0052	553	CSLK=TCK
		1/y4-2/y4	927.428	927.4216	0.0064	223	CSLK=ETCK
		1/y4-2/y5	1041.4709	1041.4377	0.0332	94	CSLK=NETCK
		1/y4-2/y6	1170.5134	1170.511	0.0024	25002	CSLK=ENETCK
		1/a1–2/y6	796.2969	796.3787	−0.0818	646	C=ENETCK
		1/b2-2/y6	911.3239	911.2963	0.0276	155	CS=ENETCK
		1/y4-2/z3	781.3588	781.3229	0.0359	77	CSLK=TCK

## 9 Disulfide bonds SS10 and SS11

**Fig. 8:**
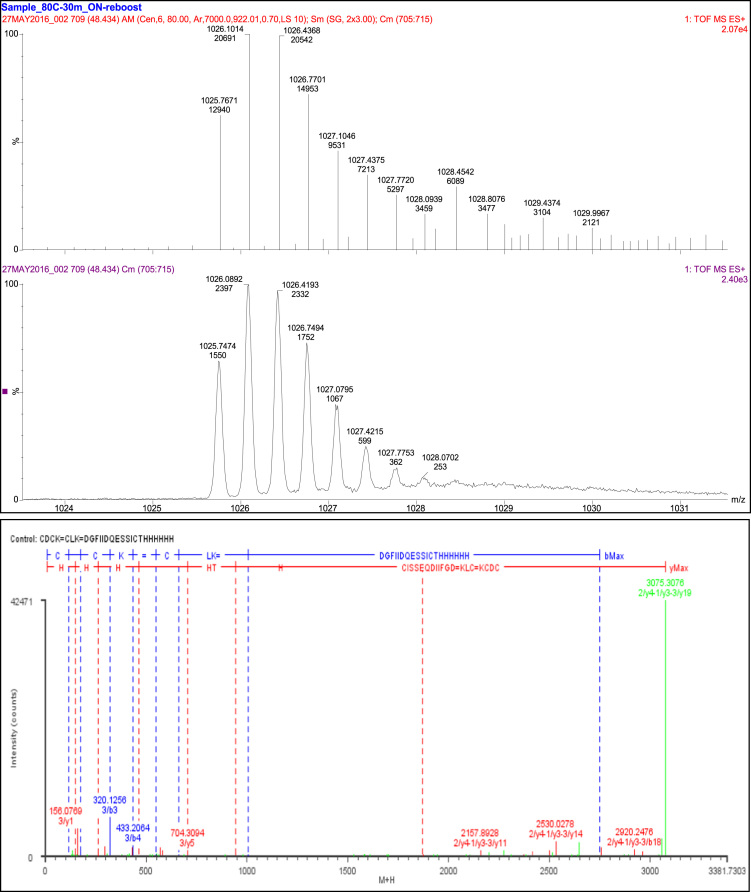
Disulfide bonds SS10 and SS11.

A total of 65 fragments were observed. Eleven fragment ions were specific to the linkage of T19 and T22, and confirmed the linkage of Cys_141_ to Cys_157_. Three fragment ions were specific to the linkage between peptides T22 and T23 and confirmed the linkage of Cys_159_ to Cys_172_. Thirty-two fragment ions were consistent with the linkage of T12, T14, and T16 and an additional 19 fragments were consistent with constituent peptides (Table 10, Fig. 8).

**Table 10:** Disulfide bond SS10 (Cys141-Cys157) and SS11 (Cys159-Cys172) peptides.

		**Assignment**	**Theoretical Mass (Da)**	**Observed Mass (Da)**	**Mass Error (Da)**	**Intensity (counts)**	**Identification**
**65 Fragments**	Constituent Peptides 19 fragments	1/y1	147.1133	147.1126	0.0007	1153	K
		1/y2	260.1974	260.2021	−0.0047	167	LK
		3/a2	145.0613	145.0646	−0.0033	400	DG
		3/a5	518.2979	518.296	0.0018	109	DGFII
		3/a8	890.426	890.3521	0.0739	84	DGFIIDQE
		3/a9	977.458	977.3666	0.0914	120	DGFIIDQES
		3/b1	116.0348	116.0532	−0.0184	28	D
		3/b2	173.0562	173.0561	0.0001	645	DG
		3/b3	320.1246	320.1256	−0.001	6533	DGF
		3/b4	433.2087	433.2064	0.0023	1906	DGFI
		3/b5	546.2927	546.2884	0.0043	325	DGFII
		3/b6	661.3197	661.3064	0.0133	295	DGFIID
		3/b9	1005.453	1005.383	0.0699	237	DGFIIDQES
		3/y1	156.0773	156.0769	0.0004	4578	H
		3/y2	293.1362	293.1368	−0.0006	1684	HH
		3/y3	430.1951	430.1913	0.0038	1430	HHH
		3/y4	567.254	567.2552	−0.0012	1460	HHHH
		3/y5	704.3129	704.3094	0.0035	1025	HHHHH
		3/y7	942.4196	942.4053	0.0143	568	THHHHHH
					
	Cys141 to Cys157 11 Fragments	1/a1–2/a1	149.0207	149.1165	−0.0958	16	C=C
		1/a2-2/a1	262.1048	262.1324	−0.0276	39	CL=C
		1/a2-2/a2	377.1317	377.1469	−0.0152	258	CL=CD
		1/a2-2/b1	290.0997	290.1243	−0.0246	45	CL=C
		1/a2-2/b2	405.1266	405.1262	0.0005	230	CL=CD
		1/y3-2/a2	551.2322	551.2063	0.0259	62	CLK=CD
		1/b1–2/a2	292.0426	292.0312	0.0114	82	C=CD
		1/b1–2/b1	205.0106	205.0966	−0.086	154	C=C
		1/b2-2/b1	318.0946	318.0872	0.0074	36	CL=C
		1/y3-2/b1	464.2001	464.214	−0.0139	41	CLK=C
		1/y3-2/b2	579.2271	579.2289	−0.0018	1027	CLK=CD
						
	Cys159 to Cys172 3 fragments	2/y2–3/a12	1527.682	1527.653	0.0291	242	CK=DGFIIDQESSIC
		2/y2–3/y19	2497.089	2497.066	0.0227	994	CK=DGFIIDQESSICTHHHHHH
		2/y3-3/a12	1642.709	1642.633	0.0763	69	DCK=DGFIIDQESSIC
						
	T12, T14, and T16 Linkage 32 Fragments	1/a1–2/a3-3/a16	2156.8411	2156.815	0.0266	37	C=CDC=DGFIIDQESSICTHHH
		1/a1–2/a3-3/y10	1609.5782	1609.559	0.0193	132	C=CDC=SICTHHHHHH
		1/a1–2/a3-3/y14	2068.7383	2068.799	−0.0608	74	C=CDC=DQESSICTHHHHHH
		1/a1–2/y4-3/y18	2673.0967	2673.159	−0.0623	62	C=CDCK=GFIIDQESSICTHHHHHH
		1/a1–2/y4-3/y8	1583.5625	1583.616	−0.0533	363	C=CDCK=CTHHHHHH
		1/a2-2/a3-3/a16	2269.925	2269.92	0.0051	921	CL=CDC=DGFIIDQESSICTHHH
		1/a2-2/a3-3/a18	2544.043	2544.035	0.0076	58	CL=CDC=DGFIIDQESSICTHHHHH
		1/a2-2/y4-3/a15	2306.9666	2306.904	0.0623	147	CL=CDCK=DGFIIDQESSICTHH
		1/b1–2/b3-3/a12	1700.6064	1700.657	−0.0505	100	C=CDC=DGFIIDQESSIC
		1/b1–2/b3-3/y14	2124.7283	2124.776	−0.0476	72	C=CDC=DQESSICTHHHHHH
		1/b1–2/y4-3/a14	2084.8186	2084.862	−0.0437	85	C=CDCK=DGFIIDQESSICTH
		1/b1–2/y4-3/y17	2644.0703	2644.117	−0.0471	2248	C=CDCK=FIIDQESSICTHHHHHH
		1/b2-2/b3-3/a12	1813.6906	1813.709	−0.0181	63	CL=CDC=DGFIIDQESSIC
		1/b2-2/b3-3/b13	1942.7332	1942.833	−0.0997	95	CL=CDC=DGFIIDQESSICT
		1/b2-2/b3-3/y8	1578.536	1578.608	−0.0721	228	CL=CDC=CTHHHHHH
		1/b2-2/b3-3/y9	1691.62	1691.693	−0.0729	105	CL=CDC=ICTHHHHHH
		1/b2-2/y4-3/a14	2197.9026	2197.909	−0.0066	671	CL=CDCK=DGFIIDQESSICTH
		1/b2-2/y4-3/b17	2637.0742	2637.017	0.0576	233	CL=CDCK=DGFIIDQESSICTHHHH
		1/b2-2/y4-3/y10	1924.7576	1924.81	−0.0524	84	CL=CDCK=SICTHHHHHH
		1/b2-2/y4-3/y16	2610.0859	2610.088	−0.0017	407	CL=CDCK=IIDQESSICTHHHHHH
		1/y3-2/y4-3/a18	2892.2439	2892.164	0.0803	403	CLK=CDCK=DGFIIDQESSICTHHHHH
		1/y3-2/y4-3/b14	2372.0032	2372.038	−0.0352	341	CLK=CDCK=DGFIIDQESSICTH
		1/y3-2/y4-3/b18	2920.2388	2920.248	−0.0088	1160	CLK=CDCK=GFIIDQESSICTHHHHHH
		1/y3-2/y4-3/y11	2157.8953	2157.893	0.0024	922	CLK=CDCK=SSICTHHHHHH
		1/y3-2/y4-3/y13	2414.9963	2415.007	−0.011	897	CLK=CDCK=QESSICTHHHHHH
		1/y3-2/y4-3/y14	2530.0232	2530.028	−0.0046	2415	CLK=CDCK=DQESSICTHHHHHH
		1/y3-2/y4-3/y16	2756.1914	2756.2	−0.0088	1421	CLK=CDCK=IIDQESSICTHHHHHH
		1/y3-2/y4-3/y18	2960.2812	2960.238	0.043	911	CLK=CDCK=GFIIDQESSICTHHHHHH
		1/y3-2/y4-3/y19	3075.3081	3075.308	0.0005	42471	CLK=CDCK=DGFIIDQESSICTHHHHHH
		1/y3-2/y4-3/y8	1870.7471	1870.768	−0.0212	131	CLK=CDCK=CTHHHHHH
		1/y3-2/y4-3/z14	2512.9968	2513.063	−0.0657	102	CLK=CDCK=DQESSICTHHHHHH
		1/y3-2/y4-3/z8	1853.7205	1853.793	−0.0724	66	CLK=CDCK=CTHHHHHH

## 10 Experimental design, materials and methods

### 10.1 Sample preparation

Baculovirus Pfs25 [2] was denatured and digested as described in [1].

### 10.2 Chromatography

Digested peptides were separated with a 2695 Separations Module (Waters Corporation; Milford MA) and a 2489 UV/Vis Detector (Waters Corporation; Milford, MA) set at 214 nm. An XBridge (Waters Corporation; Milford, MA) BEH 300 C18 (2.1×250 mm, 5 µm) was used at a column temperature of 37 °C and gradient with 0.1% Triflouroacetic acid (TFA) in purified water (Mobile Phase A) and 0.1% TFA in acetonitrile (Mobile Phase B) as described in [1].

### 10.3 Mass spectrometry

MS analysis was done with a QTOF Premier mass spectrometer (Waters Corporation; Milford, MA) equipped with an electrospray source as described in [1]. MS data was acquired in MS^E^ mode using MassLynx v4.1 (Waters Corporation; Milford, MA). RAW MS files have been deposited in the Mass Spectrometry Interactive Virtual Environment (MassIVE) with identifier: MSV000081982.

### 10.4 Analysis of mass spectra

The mass spectral data was analyzed using BiopharmaLynx 1.3 (Waters Corporation; Milford, MA) as described in [1].
